# Thyroid nodule update on diagnosis and management

**DOI:** 10.1186/s40842-016-0035-7

**Published:** 2016-10-03

**Authors:** Shrikant Tamhane, Hossein Gharib

**Affiliations:** 1 Mayo Clinic College of Medicine, Rochester, MN 55905 USA; 2 Division of Endocrinology, Diabetes, Metabolism, and Nutrition, Mayo Clinic, 200 First Street SW, Rochester, MN 55905 USA

**Keywords:** Thyroid, Thyroid nodules, Molecular markers, Benign, Malignant, FNA, Management, Ultrasonography

## Abstract

Thyroid nodules are common. The clinical importance of thyroid nodules is related to excluding malignancy (4.0 to 6.5% of all thyroid nodules), evaluate their functional status and assess for the presence of pressure symptoms. Incidental thyroid nodules are being diagnosed with increasing frequency in the recent years with the use of newer and highly sensitive imaging techniques. The high prevalence of thyroid nodules necessitates that the clinicians use evidence-based approaches for their assessment and management. New molecular tests have been developed to help with evaluation of malignancy in thyroid nodules. This review addresses advances in thyroid nodule evaluation, and their management considering the current guidelines and supporting evidence.

## Background

Thyroid nodule is a discrete lesion in the thyroid gland that is radiologically distinct from the surrounding thyroid parenchyma [1]. Thyroid nodules are common; their prevalence in the general population is high, the percentages vary depending on the mode of discovery: 2–6 % (palpation), 19–35 % (ultrasound) and 8–65 % (autopsy data) [2–4]. They are discovered either clinically on self-palpation by a patient, or during a physical examination by the clinician or incidentally during a radiologic procedure such as ultrasonography (US) imaging, computed tomography (CT) or magnetic resonance imaging (MRI) of the neck, or fluorodeoxyglucose (FDG) positron emission tomography; with the increased use of sensitive imaging techniques, thyroid nodules are being diagnosed incidentally with increasing frequency in the recent years [5, 6]. Though thyroid nodules are common, their clinical significance is mainly related to excluding malignancy (4.0 to 6.5% of all thyroid nodules) [3, 7–9], evaluating their functional status and if they cause pressure symptoms.

## Diagnosis and evaluation of thyroid nodules

Thyroid nodules can be caused by many disorders: benign (colloid nodule, Hashimoto’s thyroiditis, simple or hemorrhagic cyst, follicular adenoma and subacute thyroiditis) and malignant (Papillary Cancer, Follicular Cancer, Hurthle Cell (oncocytic) Cancer, Anaplastic Cancer, Medullary Cancer, Thyroid Lymphoma and metastases −3 most common primaries are renal, lung & head-neck) [3, 10, 11].

Initial assessment of a patient found to have a thyroid nodule either clinically or incidentally should include a detailed and relevant history plus physical examination. Laboratory tests should begin with measurement of serum thyroid-stimulating hormone (TSH). Thyroid scintigraphy/radionuclide thyroid scan should be performed in patients presenting with a low serum TSH [1]. Thyroid ultrasound should be performed in all those suspected or known to have a nodule to confirm the presence of a nodule, evaluate for additional nodules and cervical lymph nodes and assess for suspicious sonographic features. The next step in the evaluation of a thyroid nodule, if they meet the criteria as discussed later, is a fine needle aspiration (FNA) biopsy [12].

**Fig. 1:**
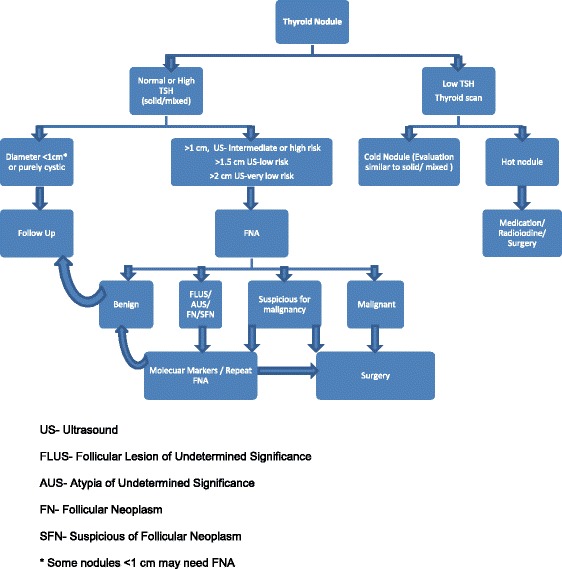
Thyroid Nodule Workup Algorithm

*Algorithm of thyroid nodule work up is presented at the end of the review (Fig. 1).

### History and physical examination

Comprehensive history with focus on risk factors predicting malignancy (Table 1 [1, 3, 13]) should be part of the initial evaluation of a patient with thyroid nodule. Symptoms of hypothyroidism or hyperthyroidism should be assessed. Patients should be questioned about local pressure symptoms such as difficulty in swallowing or breathing, cough and change in voice.

**Table 1:** Increased risk of malignancy in thyroid nodule on history and physical exam [1, 3, 13]

- History of childhood head/neck irradiation [113]	
- Total body irradiation for bone marrow transplantation [114]	
- Exposure to ionizing radiation from fallout in childhood or adolescence [115, 116]	
- Family history of PTC, MTC, or thyroid cancer syndrome (e.g., Cowden’s syndrome, familial polyposis, Carney complex, multiple endocrine neoplasia [MEN] 2, Werner syndrome) [117]	
- Enlarging nodule/rapid nodule growth	
- Cervical lymphadenopathy	
- Fixed nodule to surrounding tissue	
- Vocal cord paralysis/hoarseness

**Table 2:** Recommendations for diagnostic FNA based on size and US features [1, 35–37, 85, 86, 118–120]

A. Nodules ≥ 1 cm with intermediate or high suspicion US pattern	
B. Nodules ≥ 1.5 cm with low suspicion US pattern	
C. Nodules ≥ 2 cm with very low suspicion US pattern (e.g., spongiform). Observation an alternate option.	
D. For nodules that do not meet the above criteria, FNA is not required, including nodules < 1 cm (with some exceptions) and purely cystic nodules.	
ATA Guidelines 2015	

Physical examination focusing on the thyroid gland assessing the volume and consistency and the nodular features such as size, number, location and consistency should be performed. Thyroid nodules that are smaller, usually < 1 cm and those located posteriorly or substernally will be difficult to palpate [14, 15]. Cervical lymph nodes should be assessed. Examination of signs of hypo or hyperthyroidism should be done.

### Laboratory tests

#### Serum TSH

Serum TSH should be measured in all patients with thyroid nodules. In patients with low TSH levels, radionuclide thyroid scan should be performed next to assess the functional status of the nodule. In a patient with a thyroid nodule, an increased serum TSH or TSH even in the upper limit of normal is associated with increased risk and an advanced stage of malignancy [16, 17].

#### Serum calcitonin

In patients with thyroid nodules, the routine assessment of serum calcitonin is controversial and there are no definite recommendations for or against it [1, 18, 19]. Many prospective, non-randomized studies, mostly from outside US have assessed the value of measuring serum calcitonin [20–22]. The studies which show that use of serum calcitonin for screening may detect C-cell hyperplasia and MTC at an earlier stage and overall survival may be improved, are based on pentagastrin stimulation testing to increase specificity. Pentagastrin is not available in the United States, and there is still an ambiguity about the sensitivity/specificity, threshold cut off values and cost-effectiveness [22–24]. False-positive calcitonin results may be obtained in patients with hypercalcemia, hypergastrinemia, neuroendocrine tumors, renal insufficiency, papillary and follicular thyroid carcinomas, goiter, chronic autoimmune thyroiditis and prolonged use of certain medications [12, 25, 26]. False negative test result may be seen in rare MTCs that do not secrete calcitonin [27, 28].

#### Serum thyroglobulin (Tg)

In patients with thyroid nodules, routine measurement of serum thyroglobulin is not recommended as it can be elevated in many thyroid diseases and is neither specific nor sensitive for thyroid cancer [29, 30].

#### Serum TPO antibodies

Routine measurement of serum anti-thyroid peroxidase (TPO) antibodies is not necessary for thyroid nodule evaluation [31].

### Imaging studies

#### Radionuclide thyroid scan/scintigraphy

In patients with thyroid nodule and a low serum TSH, suggesting overt or subclinical hyperthyroidism, the next step is to determine if the nodule is autonomously functioning. Thyroid scintigraphy is useful to determine the functional status of a nodule.

Scintigraphy, a diagnostic test used in nuclear medicine, utilizing iodine radioisotopes (more commonly used; usually ^123^I) or technetium pertechnetate (^99^Tc), measures timed radioisotope uptake by the thyroid gland. The uptake of the radioisotopes will be greater in hyperfunctioning nodule and will be lower in most benign and virtually all malignant thyroid nodules than adjacent normal thyroid tissue [32–34].

Nodules may appear ‘hot’, ‘warm’ or ‘cold’ depending on whether the tracer uptake is greater than, equal to or less than the surrounding normal thyroid tissue respectively [11]. Autonomous nodules may appear hot or indeterminate and account for 5 to 10 % of palpable nodules. FNA evaluation of a hyperfunctioning nodule is not necessary as most hyperfunctioning nodules are benign [1].

#### Thyroid sonography/ultrasound

Thyroid Ultrasound (US) is a noninvasive imaging technique that should be performed on all patients with nodules suspected clinically or incidentally noted on other imaging studies such as carotid ultrasound, CT, MRI, or 18-FDG-PET scan.

Ultrasound will help confirm the thyroid nodule/s, assess the size, location and evaluate the composition, echogenicity, margins, presence of calcification, shape and vascularity of the nodules and the adjacent structures in the neck including the lymph nodes. If there are multiple nodules, all the nodules should be assessed for suspicious US characteristics.

FNA decision making is guided by both nodule size and ultrasound characteristics, the latter being more predictive of malignancy than size [35, 36]. The nodular characteristics that are associated with a higher likelihood of malignancy include a shape that is taller than wide measured in the transverse dimension, hypoechogenicity, irregular margins, microcalcifications, and absent halo [35–41]. The feature with the highest diagnostic odds ratio for malignancy was suggested to be the nodule being taller than wider [42]. The more suspicious characteristics that the nodule has, it increases the likelihood of malignancy. In contrast, benign nodule predicting US characteristics include purely cystic nodule (< 2 % risk of malignancy) [39], spongiform appearance (99.7 % specific for benign thyroid nodule) [40, 42–44].

The recent ATA guidelines classify nodules into 5 risk groups based on US results [1]. However, the current AACE guidelines suggest a more practical, 3-tier risk classification: low risk, intermediate risk and high risk thyroid lesions, based on their US characteristics [13].

In patients with thyroid nodules and low TSH who have undergone thyroid scintigraphy, ultrasound is useful to check for concordance of the nodule and hyperfunctioning area on the scan, which do not need FNA and to evaluate other nonfunctional or intermediate nodules, which may require FNA based on sonographic criteria [1].

### Fine needle aspiration biopsy (FNA)

FNA is considered the gold standard test for evaluating thyroid nodules. It is an office procedure, done under no or local anesthesia with 23 to 27 gauge needle, to obtain tissue samples for cytological examination. It is a safe, accurate and cost-effective way for evaluating thyroid nodules [45–54].

FNA can be done using palpation or with ultrasound guidance. US machines (7.5 to 10 MHz transducers), provide clear and continuous visualization of thyroid gland and allow for real time visualization of the needle tip to ensure accurate sampling. Ultrasound guided technique has lower nondiagnostic and false negative cytology rates compared to palpation technique [48, 55]. US guided FNA is preferred for difficult to palpate nodules, predominantly cystic or posteriorly located nodules [1]. In practice more clinicians are using ultrasound guided FNA (either free hand technique or with the help of a needle guide) over palpation guided technique for all thyroid FNA.

#### Indications for FNA

Over the years there has been a change in guidelines with regards to judiciously selecting the thyroid nodules for further evaluation with FNA. The approach has been toward a conservative direction. The changes are reflected in the recently published ATA guidelines [1] (Table 2).

FNA should not be performed on thyroid nodules < 1 cm in diameter with some exceptions discussed later in this section. For nodules > 1 cm, FNA is recommended to further evaluate the thyroid nodule with some exceptions [1].

FNA biopsy is recommended for nodules > 1 cm with high suspicion features (solid hypoechoic nodule or solid hypoechoic component of a partially cystic nodule with either one or more of features: irregular margin or microcalcification or taller than wide shape or rim calcification or evidence of extra thyroidal extension; estimated malignancy risk of 70–90 %) or > 1 cm with intermediate suspicion features (hypoechoic solid nodule with smooth margins without microcalcification, extra thyroidal extension or taller than wide shape; estimated malignancy risk 10–20 %). Low suspicion features include isoechoic or hyperechoic solid nodule or partially cystic nodules with eccentric solid areas without the features of highly suspicious nodule (estimated malignancy risk of 5–10 %). Cyst drainage may also be performed, especially in symptomatic patients. Very low suspicion features include spongiform (aggregation of multiple microcystic components in more than 50 % of the nodule volume [40, 52]) or partially cystic nodules without the features of the above mentioned suspicious category features (estimated malignancy risk of < 3 %).

Cervical lymph node assessment (anterior, central and lateral compartment) should be performed in all patients with thyroid nodule. Suspicious lymph nodes (microcalcification, cystic, peripheral vascularity, hyperechogenicity, round shape [56]) should have FNA evaluation for cytology and washout Tg measurement. This is one of the scenarios where a subcentimeter thyroid nodule associated with these abnormal cervical lymph nodes should undergo FNA. Also in patients with the clinical risk factors mentioned in Table 1 and with the high pretest likelihood for thyroid cancer associated with these features, FNA at sizes lower than those recommended can be considered [1, 13]. PET positive nodules have a higher incidence of malignancy ~40–45 % and FNA is recommended in nodules > 1 cm [1, 57, 58].

The US features of each nodule should be assessed independently to determine the need for FNA biopsy. The nodules that are not biopsied should be monitored with periodic US with follow up duration and frequency based on factors including sonographic features and risk factors. Also conservative approach of active surveillance without FNA may be reasonable approach for patients who meet the above FNA criteria but are at high surgical risk and those with relatively short life expectancy [1].

#### Cytological diagnosis

FNA cytology of the thyroid nodule is reported using various classification systems. In US, the Bethesda System for Reporting Thyroid Cytopathology is the most commonly used. The diagnostic groups suggested are [59, 60]:

- ➢ Benign – This includes macrofollicular or adenomatoid/hyperplastic nodules, colloid adenomas (most common), nodular goiter, lymphocytic and granulomatous thyroiditis. 0–3 % predicted risk of malignancy.
- ➢ Follicular lesion or atypia of undetermined significance (FLUS or AUS) – This includes lesions with atypical cells, or mixed macro- and microfollicular nodules. 5–15 % predicted risk of malignancy.
- ➢ Follicular neoplasm or suspicious for a follicular neoplasm (FN/SFN) – This includes microfollicular nodules, including Hurthle cell lesions/ suspicious for Hurthle cell neoplasm. 15–30 % predicted risk of malignancy.
- ➢ Suspicious for malignancy. 60–75 % predicted risk of malignancy.
- ➢ Malignant. This includes PTC (most common), MTC, anaplastic carcinoma, and high-grade metastatic cancers. 97–99 % predicted risk for malignancy.
- ➢ Nondiagnostic or Unsatisfactory. 1–4 % predicted risk of malignancy.

Other classification systems such as UK-RCPath (Royal College of Pathology) or Italian AME Consensus and modifications of these systems are also used to report cytology results [13]. The interpretation of the FNA smears is influenced by the expertise of the cytopathologist and there is inherent limitation to the reproducibility of the cytopathological results [45, 46, 61, 62]. Accuracy of the results is also influenced by the skill of the operator, FNA technique and specimen preparation. FNA results are categorized as either diagnostic/satisfactory, if it contains at least six groups, each containing of at least 10 well-preserved thyroid epithelial cells, else nondiagnostic/unsatisfactory [11, 13]. FNA results are crucial in guiding the further steps in the management of thyroid nodule.

Benign cytology (~70 % of all FNAs) is the most common finding on FNA [13, 45, 48]. Indeterminate results (~10–15 % of all FNAs), which are without a distinct cytological diagnosis [45, 46, 63], include the diagnostic groups of FLUS/AUS and FN/SFN. This diagnostic group possesses a challenge in terms of next steps for management. In practice, although the majority of these patients undergo surgery, the majority of the nodules are found benign. Molecular tests have been developed in an attempt to determine whether an indeterminate nodule is benign or malignant.

Nondiagnostic or unsatisfactory smears (~15 % of all FNAs) have inadequate number of cells to make a diagnosis and result from cystic fluid without cells, bloody smears, or improper techniques in preparing slides [11, 64–67].

### Molecular markers

The use of molecular markers in thyroid nodules has been suggested for diagnostic purpose in case of indeterminate cytological diagnosis, to assist with decision making about management option (surgical treatment). These tests are performed using samples from needle washings collected during fine needle aspiration biopsy. The molecular tests which have the most available data are: Afirma Gene-expression Classifier [68], seven-gene panel of genetic mutations and rearrangements [69] and galectin-3 immunohistochemistry [70].

The Afirma gene-expression classifier (167 GEC; mRNA expression of 167 genes) evaluates for the presence of benign gene expression profile. It has a high sensitivity (92 %) and negative predictive (93 %) value but low positive predictive value and specificity (48–53 %) [68, 71]. It is used as a rule out test to identify benign nodules. A benign GEC result predicts low risk of malignancy but the nodules classified as benign still have ~5 % risk of malignancy [71, 72].

The seven gene mutation and rearrangement analysis panel evaluates for BRAF, NRAS, HRAS and KRAS point mutations and common rearrangements of RET/PTC and PAX8/PPARγ. It has a high specificity (86–100 %) and positive predictive value (84–100 %) but poor sensitivity (reported from 44 to 100 %) [69, 73–75]. It is being used as a rule in test for thyroid malignancy.

This field is evolving and many other molecular tests are being developed (mRNA markers, miRNA markers, etc.) [70, 76–80]. None of the available tests can decisively confirm the presence or absence of malignancy in all indeterminate thyroid nodules. Long term data are needed to confirm its utility in clinical practice. Most of the assays are trained on classic papillary cancers and have limited data in follicular cancers. One has to consider performance of a diagnostic test based on prevalence of the disease (cancer); at high cancer prevalence rate, NPV falls dramatically. Tests are expensive and in deciding their use in management of indeterminate nodules, one should also consider the pretest probability of malignancy with clinical risk features, sonographic characteristics and the size of the nodule, the degree of patient concern and patient preferences, and if the patient would be able to come back for a follow up. In the current settings, molecular testing should only be used to supplement cytopathologic evaluation or clinical and imaging assessment [81]. Patients should be counselled regarding the current clinical utility and limitations of these tests. The AACE Guidelines recommend neither for nor against their use in clinical practice [13]. This field is new and evolving, the recommendations of the use of these molecular tests can be expected to change in the future.

## Management

Various factors including serum TSH, clinical risk factor assessment, size of the nodule, ultrasound characteristics, patient preferences and results of the FNA biopsy should be considered in management of thyroid nodule. FNA biopsy cytological diagnosis is the most crucial determinant in decision making.

For autonomous or hyperfunctioning nodules, if the patient has hyperthyroidism, management options include radioiodine therapy or surgery. If the patient has subclinical hyperthyroidism (low TSH with normal FT4), management depends on clinical risk of complications (atrial fibrillation in patients over the age of 60 to 65 years and osteoporosis in postmenopausal women) and the degree of TSH suppression [82–84].

Nodules less than 1 cm with some exceptions should not be biopsied and followed up closely [1]. Also for these patients the frequency and duration of follow up will depend on the additional risk factors present.

For nodules selected for FNA, management primarily depends on cytologic results. According to the Bethesda Classification scheme, FNA of the nodules yields six major results with subsequent different management for each category. However the management of indeterminate nodules (FLUS/AUS and FN/SFN) has similar principles and will be discussed together.

### Nodules with benign cytology

The risk of malignancy in nodules reported as benign is 0–3 % [85–88]. Patients with benign nodules are usually managed conservatively without surgery; immediate further diagnostic studies are not required [1]. Though there is a risk of false negative results associated with cytology reporting, initial benign FNA has negligible mortality risk in long term follow up [89].

The frequency and duration of follow up of the benign nodules have been variable in clinical practice. In the nodules that have suddenly enlarged, hemorrhage and cystic degeneration is the most common cause; malignancy is rare even in nodules that have grown [90, 91]. There is no clear evidence to suggest that nodules with larger size (> 3 or 4 cm) with benign cytology should be managed differently than smaller nodules [62, 92]. The follow up of the benign cytological diagnosis should be decided on the sonographic characteristic of the nodule rather than growth [93, 94].

Per the 2015 ATA guidelines, nodules with high suspicious US pattern should have repeat US and FNA within 12 months; while those with low to intermediate suspicious US pattern should have repeat US in 12–24 months. The decision to repeat FNA or observe with repeat US is based on > 20 % growth in at least 2 nodule dimensions or > 50 % increase in nodule volume or the appearance of new suspicious US pattern. Nodules with very low suspicious patterns should have US repeated at 24 months or more. Continued surveillance for a nodule with repeat second benign cytology is not needed [1, 95].

Surgical removal may be needed for benign nodules if they are causing pressure or structural symptoms. TSH suppressive therapy has no role in the management of benign nodule. Percutaneous ethanol ablation can be considered for thyroid cysts and certain complex thyroid nodules [13].

### Indeterminate nodules (FLUS/ AUS or FN/SFN)

FLUS/AUS and FN/SFN have 5–15 % and 15–30 % predicted risk of malignancy, respectively. Practice pattern vary considerably in management of indeterminate nodules [96]. Molecular tests have impacted the management strategies in this category. The clinical risk factors, US characteristics (Elastography in addition can be considered in these cases), patient preference and availability/feasibility of the molecular tests should be considered in the decision making process. Some scores such as Mcgill thyroid nodule scores have been tried in pre-operative decision making in thyroid nodules [97].

FLUS/AUS category includes lesions with focal architecture or nuclear atypia whose significance cannot be further determined and specimens that are limited because of poor fixation or obscuring blood [98]. The interpretation of the features which comprise this category is based entirely on the observer which results in poor reproducibility and a second review by experienced high volume cytopathologist can be considered [99, 100]. Repeat FNA or molecular testing (extra sample can be collected at the time of initial testing) can be considered to supplement the malignancy risk assessment [68, 69, 101, 102]. If either of them is not performed or inconclusive, based on clinical and US risk factors and patient preference, either surveillance with repeat US or diagnostic surgery can be chosen [1]. With the new developments in molecular testing, the approach to this category may change in the future.

For FN/SFN, surgical excision for diagnosis had been an established practice. With the molecular testing being available, it can be used to supplement the malignancy risk assessment again after considering the clinical and US risk factors and patient preference [68, 103]. If the molecular testing is not available/performed or inconclusive, diagnostic surgical excision can be considered. Patients with surgical histology specimens showing benign follicular adenoma (absence of capsular or vascular invasion) do not require further treatment. However, patients whose surgical histology shows follicular thyroid cancer might need to have a completion thyroidectomy [1, 13, 69].

### Suspicious for malignancy

This category includes specimens strongly suspicious for malignancy, but lacking diagnostic criteria [60]. Diagnostic surgery and histologic exam would be needed in most of the cases. For nodules with the cytology reported as suspicious for malignancy, after consideration of clinical and US risk factors and patient preference, molecular tests (seven-gene mutation and rearrangement panel) can be considered if it would alter the surgical decision making, which is the recommended modality of management [1, 69, 104].

As more data become available on the molecular tests, the management of this category may potentially change in the future.

### Malignant

This category includes papillary cancer, follicular carcinoma, Hurthle cell (oncocytic) carcinoma, medullary cancer, thyroid lymphoma, anaplastic cancer, and cancer metastatic to the thyroid. Surgery is generally recommended for these patients [1, 13, 105, 106]. Circumstances in which active surveillance may be considered include low risk papillary microcarcinoma (< 1 cm), patients with high surgical risk, short life expectancy and if concurrent surgical or medical issues need to be addressed first. For cancer due to metastasis, further investigations to find the primary lesion should be undertaken.

### Nondiagnostic

This category includes cytologically inadequate specimen. If no or scant follicular tissue is obtained, the absence of malignant cells does not mean a negative biopsy in patients with nondiagnostic FNA. In such cases, FNA using US-guidance should be repeated and if possible with onsite cytological assessment [1, 13, 107]. If the results are still nondiagnostic, core needle biopsy or close observation or diagnostic surgical excision can be considered depending on the suspicious pattern on sonography, clinical risk factors and growth of the nodule during active surveillance [1, 108, 109].

### Pregnancy

The evaluation of a thyroid nodule in a pregnant woman should be done in same way as one would in nonpregnant state. However, for the pregnant women with nodule and suppressed TSH that persists after first trimester, further evaluation can be delayed after pregnancy and cessation of lactation when the radionuclide scan can be performed. For a nodule with FNA suggesting PTC, if it is discovered early in pregnancy and if it grows substantially (20 % growth in at least 2 dimensions or 50 % increase in volume or new suspicious US pattern) by 24 weeks gestation or if suspicious cervical lymph nodes are noted on US, surgery should be considered during the second trimester of pregnancy. However, if it is diagnosed in the latter half of the pregnancy or if it is diagnosed early in the pregnancy and remains stable by midgestation, surgery may be performed after delivery. Consideration could be given to administration of levothyroxine therapy to keep the TSH in the range of 0.1–1 mU/L [1, 13, 110–112].

## Conclusions

Thyroid nodules are common and carry a 4–6.5 % risk of malignancy. The initial evaluation in all patients with a thyroid nodule includes a detailed history and physical examination assessing risk factors, measurement of serum TSH and neck ultrasonography to assess the size and suspicious characteristics. Fine needle aspiration (FNA) biopsy is an accurate and cost effective way to evaluate thyroid nodules. Nodules with diameter < 1 cm with some exceptions require no FNA and can be observed with a follow up US. Patients with benign nodules are usually followed without surgery. Where available, mRNA classifier system or mutational analysis can be used for further evaluating FNA aspirates with cytology of follicular neoplasm or follicular lesion/ atypia of undetermined significance. Patients with cytology suggesting cancer should be referred for surgery. The high prevalence and increasing diagnosis of incidental thyroid nodules requires clinicians to adopt evidence-based approaches to evaluate, risk stratify and provide appropriate treatment. As more evidence becomes available, active surveillance may become possible for selected cases of thyroid cancer patients.
